# Risk prediction models for malnutrition in cancer patients: a systematic review and meta-analysis

**DOI:** 10.3389/fnut.2025.1696142

**Published:** 2025-12-12

**Authors:** Jiayan Yu, Xin Chu, Dongqing Guo, Wei Luo

**Affiliations:** 1 School of Nursing, Chengdu University of Traditional Chinese Medicine, Chengdu, China; 2 Hospital of Chengdu University of Traditional Chinese Medicine, Chengdu, Sichuan, China

**Keywords:** malnutrition, cancer, risk prediction, meta-analysis, systematic review

## Abstract

**Background:**

Although numerous models have been developed in recent years to predict malnutrition in cancer patients, their methodological rigor and clinical applicability remain uncertain. The lack of systematic evaluation hampers their integration into routine oncology and nursing practice, where early identification of at-risk patients is crucial for optimizing nutritional interventions, enhancing treatment tolerance, and reducing morbidity and mortality.

**Objective:**

This systematic review aims to synthesize and critically evaluate existing risk prediction models for malnutrition in cancer patients, thereby providing evidence-based insights to inform model development and clinical implementation.

**Methods:**

Databases including PubMed, Embase, Web of Science, the Cochrane Library, and Scopus were systematically searched to identify studies on risk prediction models for malnutrition in cancer patients published from database inception to August 9, 2025. Data extracted from the included studies comprised study design, data sources, sample size, predictors, model development, and model performance. The methodological quality of each study was evaluated using the Prediction Model Risk of Bias Assessment Tool (PROBAST) checklist, and a meta-analysis of the area under the curve (AUC) was performed using Stata version 15.0.

**Result:**

A total of 13 studies encompassing 57 predictive models were included. In the model development domain, seven studies constructed models using logistic regression alone, whereas five studies combined logistic regression with machine learning techniques. The reported incidence of malnutrition ranged from 11.9 to 69.9%. The most frequently used predictors were body mass index (BMI), age, and sex. The AUC values ranged from 0.735 to 0.982, with a pooled AUC of 0.85 (95% CI: 0.79–0.92) for eight validated models, indicating good discriminative performance. All 13 studies were rated as having a high risk of bias, mainly due to inappropriate data sources and insufficient reporting within the analysis domain.

**Conclusion:**

Current models for predicting malnutrition in cancer patients remain in the exploratory phase. Although these models demonstrate good discriminatory performance, methodological shortcomings contribute to a high risk of bias. This systematic review underscores the need to integrate validated malnutrition prediction models into oncology and nursing practice. Such models can support clinicians and oncology nursing professionals in early screening and timely identification of high-risk patients, promote individualized nutritional interventions, and strengthen multidisciplinary collaboration among nurses, dietitians, and oncologists.

**Systematic review registration:**

https://www.crd.york.ac.uk/prospero/display_record.php?ID=CRD420251128218, identifier: CRD420251128218.

## 1 Background

Cancer is a disease characterized by the uncontrolled proliferation of body cells, invasion of adjacent tissues, and distant metastasis. In 2020, an estimated 19.3 million new cancer cases and nearly 10 million cancer-related deaths occurred worldwide, underscoring the increasing global cancer burden (1).

Malnutrition is highly prevalent among cancer patients, with reported rates ranging from 30 to 90% (2–4). The mechanisms underlying cancer-related malnutrition are multifactorial, involving tumor-induced mechanical obstruction, systemic metabolic disturbances, and reduced intake due to psychosocial factors and treatment-related adverse effects, such as anorexia (5–7). Malnutrition significantly affects the clinical outcomes of cancer patients. It depletes energy reserves, suppresses immune function, and impairs tissue repair, thereby increasing susceptibility to complications like infections, poor wound healing, and anastomotic fistulas (8, 9). These complications exacerbate nutritional decline, creating a vicious cycle that prolongs hospital stays and diminishes patients' quality of life (10). Additionally, malnutrition reduces tolerance to adjuvant therapies, such as chemotherapy and radiotherapy, negatively impacting treatment completion rates, survival, and long-term prognosis (11, 12). Some patients may develop cancer cachexia, a refractory metabolic syndrome resistant to conventional nutritional interventions (13). Recent studies have demonstrated that early nutritional and psychological interventions can reduce mortality risk by 32% in patients with advanced esophageal and gastric cancer (14). Therefore, developing efficient risk prediction models for early identification of high-risk individuals and personalized nutritional interventions is clinically critical for improving cancer patients' nutritional status and overall prognosis.

Given the profound impact of malnutrition on cancer treatment and prognosis, early identification through effective prediction models is paramount. These models assess multiple risk factors, assign corresponding weights, and quantify an individual's future risk, providing a scientific basis for clinical decision-making (15, 16). However, despite the increasing use of big data technology, existing malnutrition prediction models for cancer patients face several challenges, including limitations in model development quality, inconsistent predictive performance, and variable clinical applicability. Moreover, the absence of systematic comparative analyses of these models creates uncertainty regarding their clinical utility. Thus, this review aims to systematically summarize the existing malnutrition prediction models, evaluate their strengths and weaknesses, and identify key methodological gaps. By addressing these gaps, this review seeks to provide a foundation for the future development of more reliable, accurate, and clinically applicable malnutrition prediction models for cancer patients.

## 2 Methods

he study protocol was registered with PROSPERO (registration number: CRD420251128218). This review adheres to the Preferred Reporting Items for Systematic Reviews and Meta-Analyses (PRISMA) 2020 guidelines, and the PRISMA checklist is provided in the Supplementary Appendix.

### 2.1 Search strategy

A systematic search was conducted across PubMed, Embase, Web of Science, the Cochrane Library, and Scopus to identify observational studies published from database inception to August 9, 2025. A comprehensive search strategy combining subject headings and free-text terms was employed. Search terms included: Tumor^*^, Neoplas^*^, Cancer^*^, Malnourishment^*^, Undernutrition, Nutritional deficiency^*^, risk prediction, prediction model, and risk prediction model. The detailed search strategies are provided in Supplementary material (Tables A.1–A.5). Additionally, gray literature sources, including ProQuest Dissertations & Theses Global, OpenGrey, and ClinicalTrials.gov, were searched to identify unpublished or ongoing studies. Reference lists of all included studies were also reviewed to identify additional relevant publications.

This systematic review adhered to the PICOTS framework as recommended by the Critical Appraisal and Data Extraction for Systematic Reviews of Prediction Modeling Studies (CHARMS) checklist, with the aim of critically assessing and extracting data from prediction model studies (17). The PICOTS system provides a structured approach to defining the review's objective, search strategy, and inclusion/exclusion criteria for studies (18). The key components of our systematic review are outlined below:

P (Population): Cancer patients.

I (Intervention model): Risk prediction models for malnutrition in cancer patients have been developed and published (predictors ≥ 2).

C (Comparator): No competing model.

O (Outcome): The outcome focused on malnutrition rather than its subgroups.

T (Timing): The prediction of outcome is based on the comprehensive evaluation of admission information, clinical score results and laboratory test indicators.

S (Setting): The purpose of the risk prediction model is to predict the malnutrition in cancer patients, so as to promote the implementation of preventive measures to avoid adverse events.

### 2.2 Inclusion and exclusion criteria

The inclusion criteria for studies were as follows: (1) the study subjects were cancer patients; (2) the study design included prospective or retrospective cohort studies, cross-sectional studies, and case-control studies; (3) the research focused on constructing, validating, or updating predictive models for assessing malnutrition risk in cancer patients; and (4) the outcome of interest was malnutrition, as assessed by tools such as the GLIM criteria, PG-SGA scale, NRS-2002, MST, DXA, or BMI. These tools, though varying in their approaches and sensitivity, all provided a clear and validated diagnosis of malnutrition.

The exclusion criteria were: (1) studies that reported only risk factors without constructing a predictive model; (2) studies that reported prediction models with fewer than two predictors; (3) studies not published in English; (4) studies for which the full text could not be retrieved despite efforts to contact the authors via email; and (5) duplicate publications.

### 2.3 Study selection

Two researchers independently performed the initial literature screening based on the inclusion and exclusion criteria. The process involved: (1) removing duplicate records; (2) excluding studies with clearly irrelevant titles and abstracts; (3) eliminating studies unrelated to the topic through full-text review; and (4) checking the reference lists of included studies to identify additional potentially relevant literature. Any discrepancies were resolved through discussion with a third researcher to achieve consensus.

### 2.4 Data extraction

Two researchers (JYY and WL) independently extracted data following the CHARMS checklist (17). The extracted information included: data sources, study population characteristics, outcomes, predictors, sample size, statistical analysis details, predictive performance of models, and model evaluation and presentation. The data extraction process was carried out independently by both researchers with cross-verification; any discrepancies were resolved through discussion with a third researcher until consensus was reached.

### 2.5 Quality assessment

Two independent researchers systematically assessed the risk of bias and applicability of the included studies using the PROBAST (19). The tool consists of two main components: risk of bias assessment and applicability evaluation. The risk of bias assessment includes four domains (participants, predictors, outcome, and analysis) with a total of 20 signaling questions. The applicability assessment covers three domains (participants, predictors, and outcome). Each domain is rated as “low risk,” “high risk,” or “unclear risk” of bias. The overall quality assessment was as follows: if the risk of bias was low in all domains, the overall assessment was classified as “low risk”; if the risk of bias was high in any domain, the overall assessment was classified as “high risk”; and if the risk of bias was unclear in any domain while being low in others, the overall assessment was classified as “unclear.”

### 2.6 Data synthesis and statistical analysis

A meta-analysis of the AUC values for the validation models was performed using Stata software. Heterogeneity was assessed using the I^2^ index and the Q test. I^2^ values of 25%, 50%, and 75% were considered indicative of low, moderate, and high levels of heterogeneity, respectively (20). The effect model was selected based on the results of the heterogeneity tests: a fixed-effect model was used when *P* > 0.05 and *I*^2^ < 50%, while a random-effect model was applied if *P* < 0.05 or *I*^2^ ≥ 50%. To explore the sources of heterogeneity, sensitivity analysis, subgroup analysis, and meta-regression were conducted. Additionally, Egger's test was employed to assess publication bias, with *P* > 0.05 suggesting a low likelihood of publication bias (21).

## 3 Results

### 3.1 Study selection

**Figure 1:**
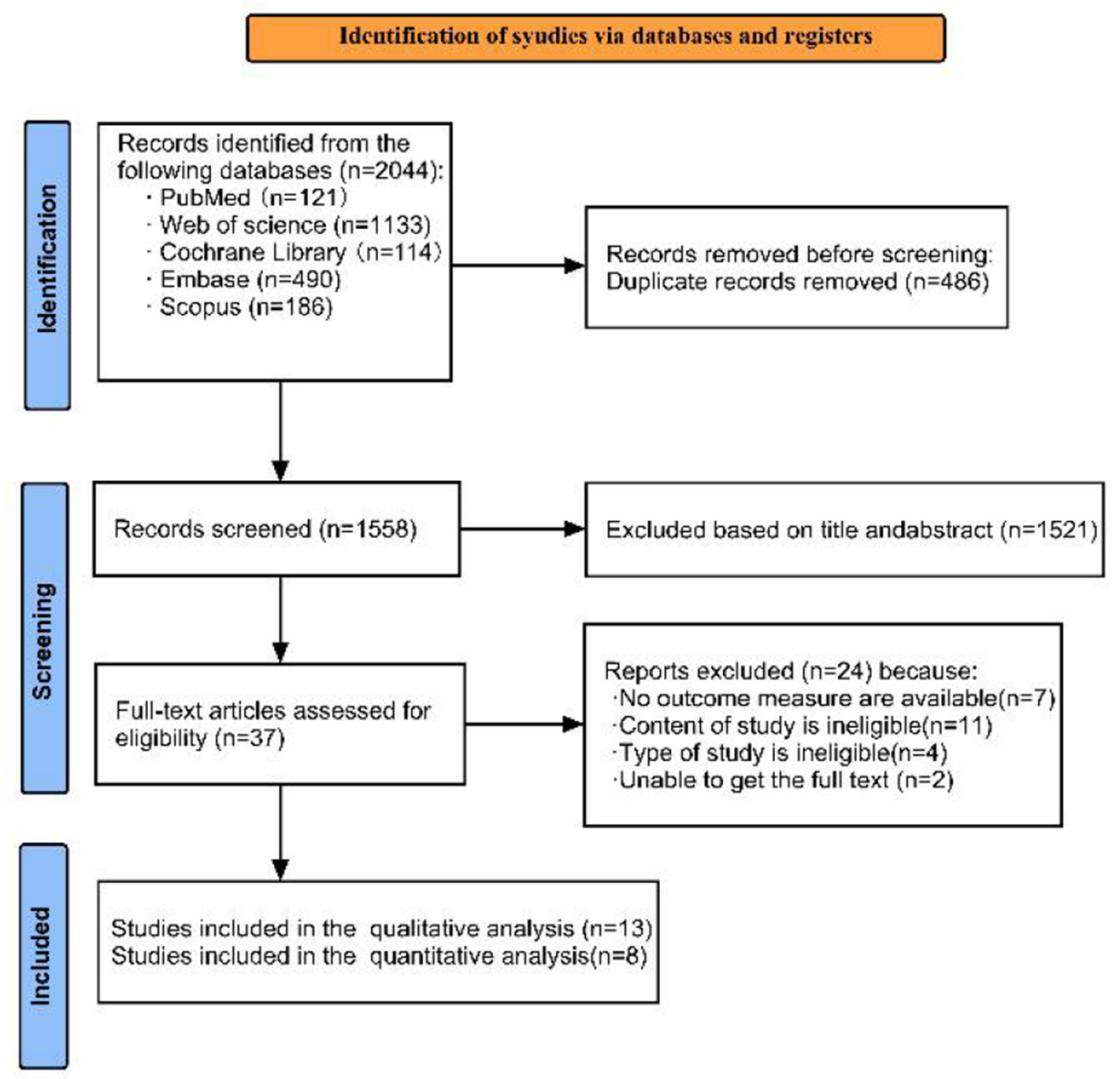
Flowchart for selection of included studies.

The study selection process is depicted in the PRISMA flow diagram (Figure 1). A total of 2,044 records were initially identified through database searches. After removing 486 duplicates, 1,558 records underwent title and abstract screening. Of these, 1,521 records were excluded as they were clearly irrelevant according to the predefined inclusion and exclusion criteria. The remaining 37 full-text articles were assessed for eligibility. Following full-text review, 24 articles were excluded for the following reasons: no relevant outcome measures (*n* = 7), ineligible study content (*n* = 11), ineligible study design (*n* = 4), and failure to retrieve the full text despite contacting the authors (*n* = 2). Consequently, thirteen studies (12, 22–33) were included in the qualitative synthesis. Of these, eight studies (22–24, 26–29, 33) with validated models were included in the quantitative synthesis (meta-analysis).

### 3.2 Characteristics of the included studies

A total of thirteen studies were ultimately included, of which twelve (12, 22–31, 33) were conducted in China and published between 2020 and 2025. Regarding study design, eleven (12, 23–32) were retrospective cohort studies, one (22) was a retrospective case-control study, and one (33) was a prospective cohort study. Three studies (24, 27, 28) were multicenter investigations, while ten (12, 22, 23, 25, 26, 29–33) were single-center studies. In terms of assessment tools, four studies (24, 26, 27, 33) used the GLIM criteria, four studies (22, 23, 30, 31) employed the PG-SGA scale, two studies (28, 29) utilized the NRS-2002, one study (12) adopted the MST, one study (25) used the DXA method, and one study (32) applied BMI. Sample sizes ranged from 120 to 4,487 participants, with outcome events occurring in 47–2,076 cases. The prevalence of malnutrition varied from 11.9% to 69.9%. Detailed study characteristics are presented in Table 1.

**Table 1:** Overview of basic data of the included studies.

**References**	**Country**	**Study design**	**Data sources**	**Participants**	**Main outcome**	**Outcome measurement tool**	**Malnutrition/sample size (%)**
Dai et al. (22)	China	Retrospective case-control study	One hospital	Gastric cancer	Malnutrition	PG-SGA	105/242 (43.4%)
Yu et al. (23)	China	Retrospective cohort study	One hospital	Cervical cancer	Malnutrition	PG-SGA	47/120 (39.2%)
Yin et al. (24)	China	Retrospective cohort study	INSCOC	Lung cancer	Malnutrition	GLIM	292/1219 (24.0%)
Yu et al. (25)	China	Retrospective cohort study	One hospital	Hepatocellular carcinoma	Malnutrition	DXA	117/220 (53.2%)
Wang et al. (26)	China	Retrospective cohort study	One hospital	Colorectal cancer	Malnutrition	GLIM	121/391 (31.0%)
Lin et al. (27)	China	Retrospective cohort study	Two hospitals	Esophageal cancer	Malnutrition	GLIM	792/1693 (46.8%)
Tang et al. (12)	China	Retrospective cohort study	One hospital	Colorectal cancer	Malnutrition	MST	149/506 (29.4%)
Wu et al. (28)	China	Retrospective cohort study	INSCOC	Colorectal cancer	Malnutrition	NRS-2002	2076/4487 (46.3%)
Huang et al. (29)	China	Retrospective cohort study	One hospital	Gastric cancer	Malnutrition	NRS-2002	142/312 (45.5%)
Duan et al. (30)	China	Retrospective cohort study	One hospital	Cancer patients	Malnutrition	PG-SGA	209/450 (46.4%)
Zhang et al. (31)	China	Retrospective cohort study	One hospital	Cancer patients	Malnutrition	PG-SGA	490/702 (69.9%)
Park et al. (32)	Korea	Retrospective cohort study	One hospital	Gastric cancer	Malnutrition	BMI	152/1281 (11.9%)
Kuang et al. (33)	China	Prospective cohort study	One hospital	Oral cancer	Malnutrition	GLIM	251/487 (51.5%)

INSCOC, investigation on nutrition status and its clinical outcome of common cancers project; DXA, dual-energy X-ray absorptiometry; PG-SGA, patient-generated subjective global assessment; GLIM, global leadership initiative on malnutrition; MST, malnutrition screening tool; BMI, body mass index.

### 3.3 Construction and validation of the model

The thirteen studies included in this review reported a total of 57 predictive models for malnutrition risk in cancer patients. Regarding predictor variables, the number of candidate variables ranged from 10 to 1,888 across the studies. Among these, seven studies (12, 22, 24–26, 32, 33) employed stepwise regression for variable selection, while the remaining studies used Lasso or machine learning methods. In terms of variable handling, five studies (12, 23, 25–27) converted continuous variables into binary categories [three of which (25–27) were based on optimal cutoff values], and eight studies (22, 24, 28–33) retained continuous variables in their original form. Eleven studies (12, 24–33) had an event per variable (EPV) ≥ 20, while two studies (22, 23) had an EPV < 20. Regarding missing data handling, only two studies (23, 30) explicitly described their methods (mean imputation and random forest imputation), one study (32) employed direct deletion, while the remaining studies did not specify their approach. Modeling approaches included: seven studies (12, 22, 24–26, 31, 32) used logistic regression alone, one study (23) combined logistic regression with Lasso, and five studies (27–30, 33) integrated logistic regression with machine learning. In terms of model validation: three studies (12, 25, 31) did not perform validation, nine studies (22–24, 26, 28–30, 32, 33) conducted internal validation only, and one study (27) performed external validation. Models were presented in various formats: eight studies (12, 22–24, 26, 27, 29, 32) used nomograms, two studies (25, 31) provided mathematical formulas, and three studies (28, 30, 33) developed online risk calculators. Detailed results are presented in Table 2.

**Table 2:** Overview of the information of the included prediction models.

**References**	**EPV**	**Missing data handling**	**Continuous variable processing method**	**Variable selection**	**Model development method**	**Validation method**	**Model performance**	**Calibration method**	**Model presentation**	**Final predictors**
Dai et al. (22)	< 20	–	Continuous variable	Backwards stepwise regression	LR	Internal validation: Bootstrap Random splitting	A: 0.840 (0.787–0.884) B1: 0.854 (0.770–0.916)	Calibration curve Hosmer–Lemeshow Brier score	Nomogram	TNM CFG PAB NLR EN within 48 h post-op
Yu et al. (23)	< 20	Mean substitution	Categorical variables	LASSO Backwards stepwise regression	LASSO LR	Internal validation: Random splitting	Model1 (radiomics model) A: 0.778 (0.339–1.000) B1: 0.776 (0.623–0.930) Model2 (clinical model) A: 0.847 (0.557–1.000) B1: 0.776 (0.607–0.946) Model3 (combined model) A: 0.972 (0.895–1.000) B1: 0.805 (0.713–0.996)	Calibration curve	Nomogram	Rad score Age ECOG PS
Yin et al. (24)	≥20	–	Continuous variable	Both directions stepwise regression	LR	Internal validation: Bootstrap Random splitting	A: 0.974 (0.962–0.985) B1: 0.982 (0.969–0.995)	Calibration curve Hosmer–Lemeshow	Nomogram	Sex BMI Weight loss within 6 month Weight loss beyond 6 month Calf circumference Handgrip strength/weight ratio
Yu et al. (25)	≥20	–	Categorical variables (Optimal cut of)	Stepwise regression	LR	–	Model1 A: 0.990 (0.966–0.997) Model2 (simplified model) A: 0.986 (0.961–0.997)	Hosmer–Lemeshow	Formula of risk score obtained by partial regression coefficient of each factor	Age Tumor diameter TNM staging Anemia
Wang et al. (26)	≥20	–	Categorical variables (Optimal cut of)	Forward stepwise regression	LR	Internal validation: Bootstrap	B1: 0.958 (0.937–0.979)	Calibration curve Hosmer–Lemeshow	Nomogram	NRS-2002 Prolonged bed rest BMI PNI
Lin et al. (27)	≥20	–	Categorical variables (Optimal cut of)	LASSO Least absolute shrinkage	LR Machine learning	External validation	**LR** A: 0.795 (0.764–0.828) B2: 0.801 (0.775–0.826) **RF** A: 0.820 (0.796–0.845) B2: 0.805 (0.771–0.839) **KNN** A: 0.760 (0.734–0.787) B2: 0.795 (0.760–0.829) **GNB** A: 0.659 (0.629–0.690) B2: 0.790 (0.756–0.825) **PLS** A: 0.669 (0.639–0.699) B2: 0.753 (0.715–0.791) **NN** A: 0.620 (0.589–0.652) B2: 0.773 (0.737–0.808) **TB** A: 0.688 (0.659–0.718) B2: 0.761 (0.725–0.797) **EGB** A: 0.740 (0.712–0.768) B2: 0.755 (0.718–0.792) **SVM** A: 0.636 (0.606–0.667) B2: 0.735 (0.698–0.773)	Calibration curve Hosmer–Lemeshow	Nomogram	Gender Age Preoperative MBI Neoadjuvant therapy history Preoperative sarcopenia
Tang et al. (12)	≥20	–	Categorical variables	Stepwise regression	LR	–	A: 0.745 (0.697–0.793)	Calibration curve Hosmer–Lemeshow	Nomogram	Age BMI ECOG PS Intention of treatment Albumin Fatigue Change in stool
Wu et al. (28)	≥20	–	Continuous variable	“Caret” package	LR Machine learning	Internal validation: Random splitting	Full models (44 predictors) **LR** A: 0.803 (0.788–0.818) B1: 0.796 (0.769–0.822) **DT** A: 0.814 (0.800–0.828) B1: 0.801 (0.775–0.827) **AdaBoost** A: 0.882 (0.870–0.893) B1: 0.840 (0.817–0.864) **RF** A: 1.000 (1.000–1.000) B1: 0.836 (0.812–0.859) **SVM** A: 0.889 (0.878–0.901) B1: 0.807 (0.781–0.832) **NNET**A: 0.714 (0.698–0.730) B1: 0.719 (0.692–0.746) Simplified models (14 predictors) **LR** A: 0.799 (0.784–0.814) B1: 0.794 (0.767–0.820) **DT** A: 0.812 (0.789–0.826) B1: 0.805 (0.780–0.830) **AdaBoost** A: 0.853 (0.840–0.866) B1: 0.824 (0.799–0.849) **RF** A: 1.00 (1.000–1.000) B1: 0.830 (0.805–0.854) **SVM** A: 0.857 (0.844–0.870) B1: 0.809 (0.784–0.835) **NNET** A: 0.657 (0.641–0.673) B1: 0.665 (0.637–0.692) Simplified models (8 predictors) **RF**B1: C-index: 0.782 (0.754–0.810)	–	Online risk calculator	BMI Age HGS MAMC HDL Triglyceride Hemoglobin Prealbumin
Huang et al. (29)	≥20	–	Continuous variable	PCA LASSO Stepwise regression	LR Machine learning	Internal validation: Random splitting Cross validation	Machine learning (CT image deep-learning feature model) **NB** B1: 0.751 (0.651–0.851) **SVM** B1: 0.738 (0.638–0.837) **KNN** B1: 0.580 (0.466–0.693) **RF** B1: 0.672 (0.563–0.780) **Extra Trees** B1: 0.657 (0.549–0.765) **XGBoost** B1: 0.703 (0.595–0.810) **Light GBM** B1: 0.680 (0.569–0.791) **GB** B1: 0.683 (0.574–0.791) **AB** B1: 0.714 (0.609–0.817) **MLP** B1: 0.769 (0.673–0.863) Mixed model (combines deep learning features and clinical factors) A: 0.909 (0.869–0.948) B1: 0.847 (0.782–0.931)	Calibration curve	Nomogram	BMI Lymphocyte Albumin DL-score
Duan et al. (30)	≥20	Random forest filling	Continuous variable	LASSO	LR Machine learning	Internal validation: Random splitting	**LR** A: 0.885 B1: 0.863 **XGBoost** A: 0.984 B1: 0.945 **RF** A: 1.000 B1: 0.937 **GNB** A: 0.886 B1: 0.881 **CNB** A: 0.870 B1: 0.850 **SVM** A: 0.991 B1: 0.894 **KNN** A: 1.000 B1: 0.901 **AdaBoost** A: 0.956 B1: 0.924 **MLP** A: 0.867 B1: 0.875	Calibration curve	Nomogram Web-based tool	ADL ALB BMI Age
Zhang et al. (31)	≥20	–	Continuous variable	Stepwise Regression DT RF	LR	–	A: 0.813	Calibration curve	Formula of risk score obtained by partial regression coefficient of each factor	Age Tumor type BMI PA-LA
Kuang et al. (33)	≥20	–	Continuous variable	Stepwise regression	LR Machine learning	Internal validation: Random splitting	**LR** A: 0.778 (0.730–0.827) B1: 0.788 (0.715–0.861) **LGBM** A: 0.751 (0.700–0.802) B1: 0.771 (0.695–0.847) **SVM** A: 0.776 (0.727–0.825) B1: 0.779 (0.705–0.853) **XGB** A: 0.872 (0.836–0.909) B1: 0.840 (0.777–0.904)	Calibration curve	Web calculator	Sex TNM Repair and reconstruction Diabetes status Age Lymphocyte countt Total cholesterol

“–”, not reported; A, development cohort; B1, internal validation cohort; B2, external validation cohort; LASSO, least absolute shrinkage and selection operator; LR, logistic regression; RF, random forest; KNN, K-Nearest Neighbor; GNB, Gaussian Naive Bayes; PLS, partial least squares; NN, neural network; TB, Tree Bagger; EGB, extreme gradient boosting; SVM, support vector machine; DT, decision tree; NNET, neural network; NB, Naïve Bayes; MLP, multilayer perceptron; CNB, complement Naive Bayes; GB, gradient boosting; LGBM, light gradient boosting machine; TNM, tumor node metastasis; CFG, cardiac function grading; NLR, neutrophil-to-lymphocyte ratio; PAB, prealbumin; ECOG PS, eastern cooperative oncology group performance status; BMI, body mass index; PNI, prognostic nutritional index; HGS, hand grip strength; MAMC, mid-arm muscle circumference; HDL, high-density lipoprotein; ADL, activities of daily living; ALB, albumin; PA-LA, PA of the left arm.

### 3.4 Prediction model performance

Given that some studies developed multiple models based on the same sample using different predictors, only data from their final combined or simplified models were included for performance evaluation. The results of the model performance analysis are as follows: regarding model discrimination, eleven studies (12, 22–25, 27, 28, 30–33) reported AUC values (0.620–1.000) during the modeling phase. Nine studies (22–24, 26, 28–30, 32, 33) provided internal validation AUC values (0.771–0.982), and one study (27) reported external validation AUC values (0.735–0.805). In terms of calibration assessment, six studies (23, 29–33) presented results solely through calibration curves, one study (25) reported only Hosmer–Lemeshow test results, and four studies (12, 24, 26, 27) provided both Hosmer–Lemeshow test results and calibration curves. One study (22) reported calibration curves, Hosmer–Lemeshow test results, and Brier score data, while one study (28) did not perform a calibration analysis. Detailed results are presented in Table 2.

### 3.5 Meta-analysis of validation models included in the review

**Figure 2:**
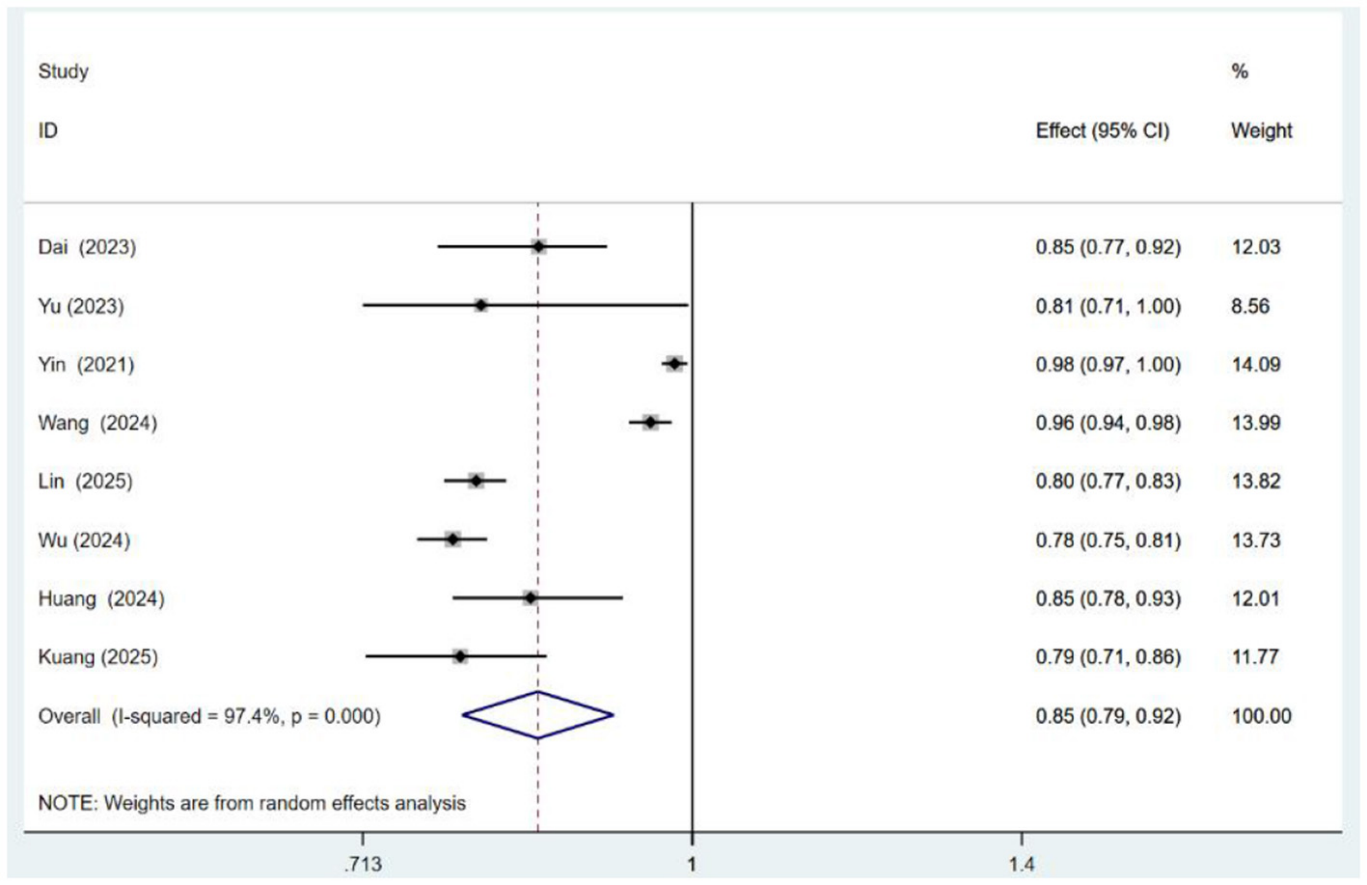
Forest plot of a risk prediction model for malnutrition in cancer patients.

Due to insufficient reporting of model development details, only eight studies (22–24, 26–29, 33) met the criteria for meta-analysis. Among these, the studies by Lin et al. (27) and Kuang et al. (33) used different modeling methods based on the same sample, so only the logistic regression (LR) models were included. For studies with multiple models using different predictors (23, 25, 28, 29), only data from simplified or combined models were pooled. The meta-analysis revealed substantial heterogeneity (*I*^2^ = 97.4%, *p* < 0.001). To explore the sources of this heterogeneity, we conducted a sensitivity analysis, which showed that the pooled AUC remained stable, with no single study unduly influencing the result (Supplementary Figure S1). Subgroup analyses based on the diagnostic tool for malnutrition and meta-regression with continuous variables (sample size, prevalence of malnutrition, and publication year) were also performed. However, none of these analyses identified a statistically significant source that could adequately explain the high heterogeneity. Therefore, a random-effects model was adopted, and the pooled AUC was 0.85 (95% CI: 0.79–0.92). An AUC value between 0.8 and 0.9 indicates good predictive performance and stability. Egger's test suggested no significant publication bias (*P* = 0.078), and the funnel plots are shown in Supplementary Figures S2. The meta-analysis results are presented in Figure 2.

### 3.6 Risk of bias and applicability evaluation

Table 3 presents the risk of bias and applicability assessment results for the included studies. In the participants domain, twelve studies (12, 22–32) were classified as high risk of bias, as all were retrospective studies. None of these data sources adhered to the low-bias data sources recommended by the PROBAST guidelines, such as prospective cohort studies, randomized controlled trials, or registry databases. In the predictors domain, one study (22) was rated as high risk of bias because it was a case-control study that assessed predictors after the outcomes were known. In the outcome domain, two studies (25, 32) which used BMI and DXA as diagnostic criteria, were rated as high risk of bias. In the analysis domain, all studies were assessed as high risk. Specific reasons included: two studies (22, 23) with EPV ≤ 20; ten studies (12, 22, 24–29, 31, 33) that did not describe their methods for handling missing data; and seven studies (12, 22, 24–26, 32, 33) that selected predictive variables based solely on univariate analysis. Regarding model validation, three studies (12, 25, 31) developed models without validation, while nine studies (22–24, 26, 28–30, 32, 33) performed only internal validation. In terms of applicability, only two studies (25, 32), which used BMI and DXA as diagnostic criteria, demonstrated low applicability, while the remaining studies showed good applicability.

**Table 3:** PROBAST results of the included studies.

**References**	**ROB**				**Applicability**			**Overall**	
	**Participants**	**Predictors**	**Outcome**	**Analysis**	**Participants**	**Predictors**	**Outcome**	**ROB**	**Applicability**
Dai et al. (22)	–	–	+	–	+	+	+	–	+
Yu et al. (23)	–	+	+	–	+	+	+	–	+
Yin et al. (24)	–	+	+	–	+	+	+	–	+
Yu et al. (25)	–	+	–	–	+	+	–	–	–
Wang et al. (26)	–	+	+	–	+	+	+	–	+
Lin et al. (27)	–	+	+	–	+	+	+	–	+
Tang et al. (12)	–	+	+	–	+	+	+	–	+
Wu et al. (28)	–	+	+	–	+	+	+	–	+
Huang et al. (29)	–	+	+	–	+	+	+	–	+
Duan et al. (30)	–	+	+	–	+	+	+	–	+
Zhang et al. (31)	–	+	+	–	+	+	+	–	+
Park et al. (32)	–	+	–	–	+	+	–	–	–
Kuang et al. (33)	+	+	+	–	+	+	+	–	+

PROBAST, prediction model risk of bias assessment tool; ROB, risk of bias.

+ indicates low ROB/low concern regarding applicability; – indicates high ROB/high concern regarding application.

## 4 Discussion

### 4.1 The overall performance of the risk prediction model for malnutrition in cancer patients is good, but the risk of bias is relatively high

The thirteen studies included in this review reported a total of 57 predictive models, all demonstrating strong performance, with AUC values ranging from 0.735 to 0.982. The pooled AUC for eight validated models was 0.85 (95% CI: 0.79–0.92), reflecting excellent discriminatory power. Additionally, the calibration curves, Hosmer–Lemeshow (H–L) test, and Brier score results collectively confirmed satisfactory model calibration. A key finding of our meta-analysis is the substantial, unresolved heterogeneity (*I*^2^ = 97.4%). Despite extensive investigations, we were unable to identify definitive sources of this heterogeneity. This significant limitation warrants caution when interpreting the pooled AUC. The heterogeneity likely arises from a combination of factors, including clinical diversity (e.g., varying cancer types and malnutrition mechanisms), differences in diagnostic criteria (e.g., PG-SGA, GLIM), and methodological variations in model development and validation. As such, our pooled estimate should be considered a summary of a heterogeneous evidence base, highlighting the early stage of this field and underscoring the urgent need for standardized outcome definitions in future research.

According to the PROBAST tool, all included studies exhibited a high risk of bias, particularly in the domains of study design and analysis, which warrants caution in applying these models in clinical practice. Regarding the study subjects, all twelve studies included in this review employed retrospective designs. These studies relied on pre-existing data, which may contain missing information or recording errors, leading to recall bias, information bias, and selection bias. Additionally, since predictor variables were collected after the outcome, these studies have limited ability to establish causal relationships. In contrast, prospective studies, which collect predictors before the outcome, offer improved data reliability and greater accuracy in predictive models. According to the PROBAST tool, suitable study designs include prospective cohort studies, randomized controlled trials, nested case-control studies, or case-cohort studies (19). In terms of analysis, nine studies did not describe how they handled missing values, which may introduce selection bias and affect the accuracy and reliability of the models (34). Common methods for handling missing data include complete-case deletion, multiple imputation, and machine learning techniques. While complete-case deletion can reduce sample size and limit dataset diversity, multiple imputation and machine learning methods use more data and improve statistical power (34, 35). Proper reporting and handling of missing data are essential to prevent model overfitting (36). Future studies should improve missing data management to enhance research completeness and credibility. Six studies relied solely on univariate analysis for variable selection, identifying predictors that were significant individually. This approach overlooked interactions among variables, potentially excluding important predictors and leading to overfitting, which reduces predictive performance (19). Current research suggests that methods like LASSO, Ridge, Elastic Net regression, and machine learning can improve variable selection accuracy (37). Future studies should adopt these methods and consider clinical knowledge, predictor reliability, and applicability in the selection process, rather than relying solely on univariate analysis results (38). Internal validation is used to assess model reproducibility and prevent overfitting, while external validation evaluates a model's generalizability and clinical applicability, which is considered the “gold standard” (39, 40). In this study, four studies lacked internal validation, and only one performed external validation. Most internal validations used simple data splitting, which can be ineffective with small sample sizes and may lead to biased results. It is recommended to use more robust methods, such as cross-validation or bootstrapping, to improve model stability (41). Furthermore, most studies lacked external validation, and the research populations were mainly from single-center studies in China, limiting model generalizability. Future research should strengthen external validation by including diverse populations from different regions, ethnicities, cultures, and lifestyles. Multicenter studies are also encouraged to reduce biases associated with single-center data.

### 4.2 BMI age and sex are common predictors

The predictive models in this study included 3–8 predictors, with BMI (*n* = 6), age (*n* = 5), and gender (*n* = 4) being the most common. These factors are crucial for both clinical practice and future research. BMI was the most frequently identified predictor, consistent with findings from previous studies (42–44), and is a key parameter in nutritional screening tools (45). A low BMI indicates insufficient nutritional reserves, which, when combined with surgery and cancer treatments, increases the risk of malnutrition (27). It also impairs the body's ability to absorb nutrients, exacerbating malnutrition (46). Age is another consistent risk factor for malnutrition in cancer patients, as aging affects nutrient metabolism and absorption, while chronic diseases and medications further compromise nutrition (12, 23, 25, 27, 33). Limited mobility and psychological factors can also reduce food intake (47–49). Therefore, healthcare providers should focus on elderly patients with low BMI to prevent malnutrition. Gender showed inconsistent findings: two studies (27, 32) found a higher risk of malnutrition in women, while two others (24, 33) indicated that men were more susceptible. This variability may be due to differences in cancer types, treatments, and socio-cultural factors. Future research should explore how sex interacts with other risk factors in different cancer populations and contexts. Other predictors, such as the ECOG score (12, 23), albumin level (12, 29), and radiological indicators (23, 29), were also identified and could enhance predictive models. Future studies should validate these predictors in diverse populations and clinical settings to develop more reliable models for malnutrition.

### 4.3 Implications for future model research

With the rapid advancement of big data and artificial intelligence, computational algorithms are increasingly integrated into clinical medicine. Studies by Kuang et al. (33), Lin et al. (27), Wu et al. (28), and Duan et al. (30) developed prediction models using both traditional logistic regression and machine learning algorithms. The latter two studies demonstrated that machine learning models often outperformed logistic regression, although the differences in AUC were small. Logistic regression is simple, interpretable, and performs well with small datasets but struggles with nonlinear relationships, missing values, and multicollinearity (50). Machine learning, on the other hand, handles large-scale, high-dimensional data more effectively and addresses these challenges, offering greater robustness (51). However, machine learning models are more complex, harder to interpret, and vulnerable to issues during modeling (52). Therefore, careful algorithm selection and tuning are critical to ensuring model reliability. In conclusion, no single method is inherently superior; performance may vary across different datasets and domains. Future research should focus on optimizing machine learning algorithms and comparing various approaches to identify the best-performing model.

## 5 Limitations

This systematic review has several limitations: (1) only studies published in Chinese and English were included, which may introduce a risk of language bias. (2) The majority of the studies were single-center investigations conducted in China, which lack external validation and limit the generalizability of the results to other regions. (3) Despite thorough examination of the high heterogeneity in AUC values through sensitivity analysis, subgroup analysis, and meta-regression, the underlying causes of this variation remain unclear. This limitation compromises the reliability and generalizability of the pooled estimates, reflecting the substantial methodological and clinical diversity in the current literature. (4) Due to significant heterogeneity in data types, definitions, and cutoff values among the included predictive factors, and the fact that most factors were only reported in a single study, a meta-analysis could not be conducted. As a result, this review focused on providing a systematic synthesis of the relevant predictive factors. (5) While most models demonstrated strong predictive performance, the high risks associated with the data sources and analytical methods used in model development necessitate careful evaluation and selection of models by clinicians or oncology nursing professionals. Further validation of model stability is recommended.

## 6 Conclusion

This review systematically summarizes existing malnutrition prediction models. While current models demonstrate promising predictive performance, their methodological limitations, including issues with study design, data handling, and model validation, introduce significant risks of bias. Addressing these gaps is crucial for improving both the accuracy and clinical applicability of these models. Future research should adhere to PROBAST guidelines, focusing on large-scale, multicenter prospective studies and incorporating machine learning techniques to optimize model development. This approach aims to construct well-performing and stable models. By improving the identification of at-risk cancer patients, this research offers valuable insights for oncology nursing practice, enabling clinicians and oncology nursing professionals to implement more targeted and timely nutritional interventions, ultimately enhancing patient outcomes.

## Data Availability

The original contributions presented in the study are included in this article/Supplementary material, further inquiries can be directed to the corresponding author.
