# Supplementary material for: Risk prediction models for malnutrition in cancer patients: a systematic review and meta-analysis
Source: Front Nutr. 2025 Dec 12;12:1696142. doi: 10.3389/fnut.2025.1696142 (PMC12740756; doi:10.3389/fnut.2025.1696142)

**Supplementary material**

**1. Supplementary Tables**

**Supplementary Table A.1.** PubMed (August 9, 2025)

| **Search** | **Query** | **Items found** |
| --- | --- | --- |
| #1 | **Neoplasms[MeSH Terms]** | 4139638 |
| #2 | **(Tumor*[Title/Abstract]) OR (Neoplas*[Title/Abstract]) OR (Cancer*[Title/Abstract]) OR (Malignanc*[Title/Abstract]) OR (Malignant Neoplasm*[Title/Abstract]) OR (Benign Neoplasm*[Title/Abstract])** | 4006878 |
| #3 | **#1 OR #2** | 5419977 |
| #4 | **Malnutrition[MeSH Terms]** | 142129 |
| #5 | **(Malnourishment*[Title/Abstract]) OR (Undernutrition[Title/Abstract]) OR (Nutritional Deficien*[Title/Abstract]) OR (undernourishment*[Title/Abstract])** | 19729 |
| #6 | **#4 OR #5** | 155137 |
| #7 | **(risk prediction[Title/Abstract]) OR (prediction model[Title/Abstract]) OR (risk prediction model[Title/Abstract]) OR (prognostic model[Title/Abstract]) OR (prediction tool[Title/Abstract]) OR (risk Score[Title/Abstract]) OR (risk assessment[Title/Abstract]) OR (Nomogram[Title/Abstract])** | 226262 |
| #8 | **#3 AND #6 AND #7** | 121 |

**Supplementary Table A.2.** Embase(August 9, 2025)

| **Search** | **Query** | **Items found** |
| --- | --- | --- |
| #1 | 'neoplasm'/exp | 6836030 |
| #2 | 'tumor*':ti,kw,ab OR 'neoplas*':ti,kw,ab OR 'cancer*':ti,kw,ab OR 'malignanc*':ti,kw,ab OR 'malignant neoplasm*':ti,kw,ab OR 'benign neoplasm*':ti,kw,ab | 5664555 |
| #3 | **#1 OR #2** | 7906758 |
| #4 | 'malnutrition'/exp | 235461 |
| #5 | 'malnourishment*':ti,kw,ab OR 'undernutrition':ti,kw,ab OR 'nutritional deficien*':ti,kw,ab OR 'undernourishment*':ti,kw,ab | 27672 |
| #6 | #4 OR #5 | 248696 |
| #7 | 'risk prediction':ti,kw,ab OR 'prediction model':ti,kw,ab OR 'risk prediction model':ti,kw,ab OR 'prognostic model':ti,kw,ab OR 'prediction tool':ti,kw,ab OR 'risk score':ti,kw,ab OR 'risk assessment':ti,kw,ab OR 'nomogram':ti,kw,ab | 309433 |
| #8 | #3 AND #6 AND #7 | 490 |

**Supplementary Table A.3.** Cochrane(August 9, 2025)

| **Search** | **Query** | **Items found** |
| --- | --- | --- |
| #1 | MeSH descriptor: [Neoplasms] explode all trees | 128996 |
| #2 | (Tumor*):ti,kw,ab OR (Neoplas*):ti,kw,ab OR (Cancer*):ti,kw,ab OR (Malignanc*):ti,kw,ab OR (Malignant Neoplasm*):ti,kw,ab OR (Benign Neoplasm*):ti,kw,ab | 281142 |
| #3 | #1 OR #2 | 295090 |
| #4 | MeSH descriptor: [Malnutrition] explode all trees | 6214 |
| #5 | (Malnourishment*):ti,kw,ab OR (Undernutrition):ti,kw,ab OR (Nutritional Deficien*):ti,kw,ab OR (undernourishment*):ti,kw,ab | 3921 |
| #6 | #4 OR #5 | 9203 |
| #7 | (risk prediction):ti,kw,ab OR (prediction model):ti,kw,ab OR (risk prediction model):ti,kw,ab OR (prognostic model):ti,kw,ab OR (prediction tool):ti,kw,ab OR (risk Score):ti,kw,ab OR (risk assessment):ti,kw,ab OR (Nomogram):ti,kw,ab | 201961 |
| #8 | #3 AND #6 AND #7 | 114 |

**Supplementary Table A.4.** WOS(August 9, 2025)

| **Search** | **Query** | **Items found** |
| --- | --- | --- |
| **#1** | **AB=(Tumor* OR Neoplas* OR Cancer* OR Malignanc* OR Malignant Neoplasm* OR Benign Neoplasm*)** | 1994384 |
| **#2** | **AB=(Malnourishment* OR Undernutrition OR Nutritional Deficien* OR undernourishment* OR Malnutrition)** | 41248 |
| **#3** | **AB=(risk prediction OR prediction model OR risk prediction model OR prognostic model OR prediction tool OR risk Score OR risk assessment OR Nomogram)** | 1121577 |
| **#4** | **#1 AND #2 AND #3** | 1133 |

**Supplementary Table A.5.** Scopus (August 9, 2025)

| **Search** | **Query** | **Items found** |
| --- | --- | --- |
| #1 | ( ABS ( "Tumor*" ) OR ABS ( "Neoplas*" ) OR ABS ( "Cancer*" ) OR ABS ( "Malignanc*" ) OR ABS ( "Malignant Neoplasm*" ) OR ABS ( "Benign Neoplasm*" ) ) | 4280361 |
| #2 | ( ABS ( "Malnourishment*" ) OR ABS ( "Undernutrition" ) OR ABS ( "Nutritional Deficien*" ) OR ABS ( "undernourishment*" ) OR ABS ( "Malnutrition" ) ) | 88693 |
| #3 | ( ABS ( "risk prediction" ) OR ABS ( "prediction model" ) OR ABS ( "risk prediction model" ) OR ABS ( "prognostic model" ) OR ABS ( "prediction tool" ) OR ABS ( "risk Score" ) OR ABS ( "risk assessment" ) OR ABS ( "Nomogram" ) ) | 469207 |
| #4 | ((ABS ( "Tumor*" ) OR ABS ( "Neoplas*" ) OR ABS ( "Cancer*" ) OR ABS ( "Malignanc*" ) OR ABS ( "Malignant Neoplasm*" ) OR ABS ( "Benign Neoplasm*" ) ) AND ( ABS ( "Malnourishment*" ) OR ABS ( "Undernutrition" ) OR ABS ( "Nutritional Deficien*" ) OR ABS ( "undernourishment*" ) OR ABS ( "Malnutrition" ) ) AND ( ABS ( "risk prediction" ) OR ABS ( "prediction model" ) OR ABS ( "risk prediction model" ) OR ABS ( "prognostic model" ) OR ABS ( "prediction tool" ) OR ABS ( "risk Score" ) OR ABS ( "risk assessment" ) OR ABS ( "Nomogram" ))) | 186 |

**2. Supplementary Figures**

**Supplementary Figure S1.** Sensitivity Analysis Plot


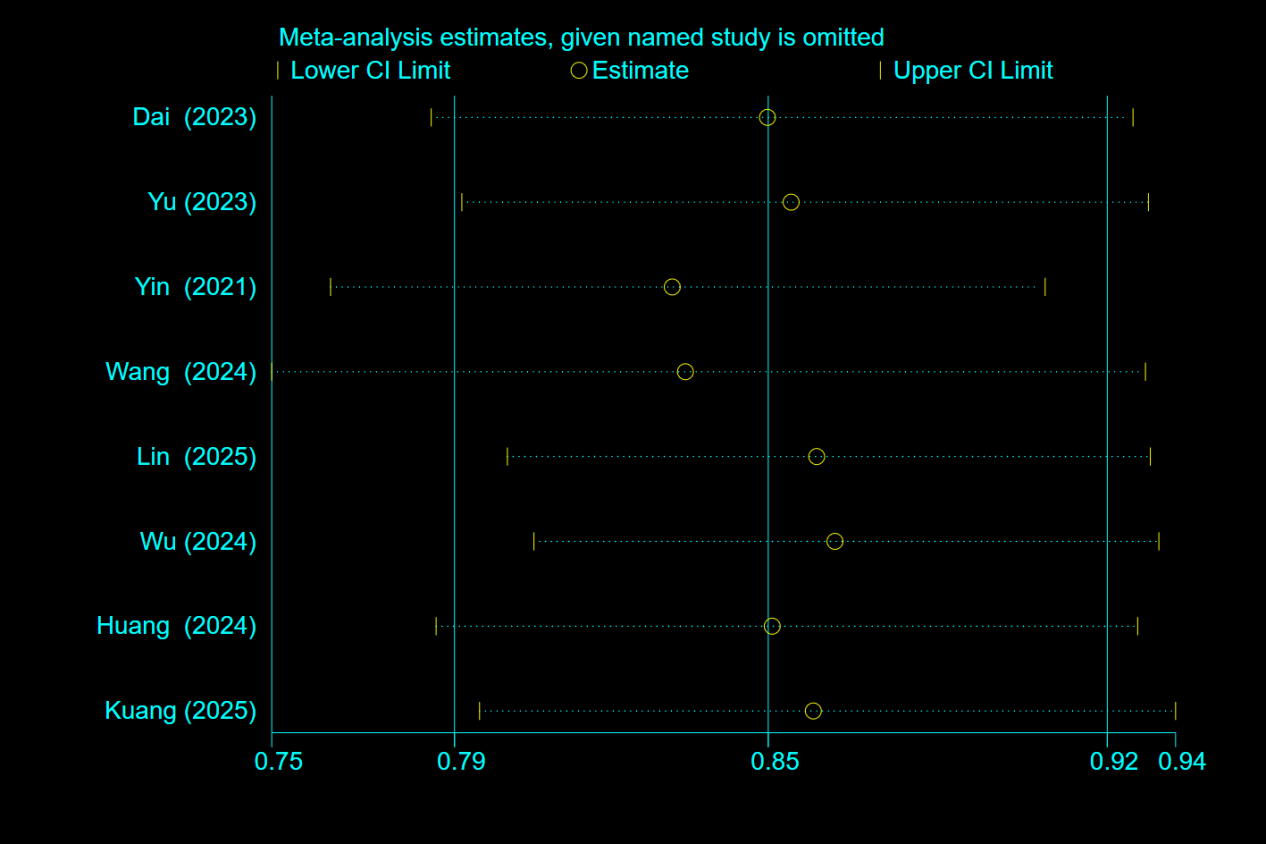


**Supplementary Figure S2.** Funnel Plot


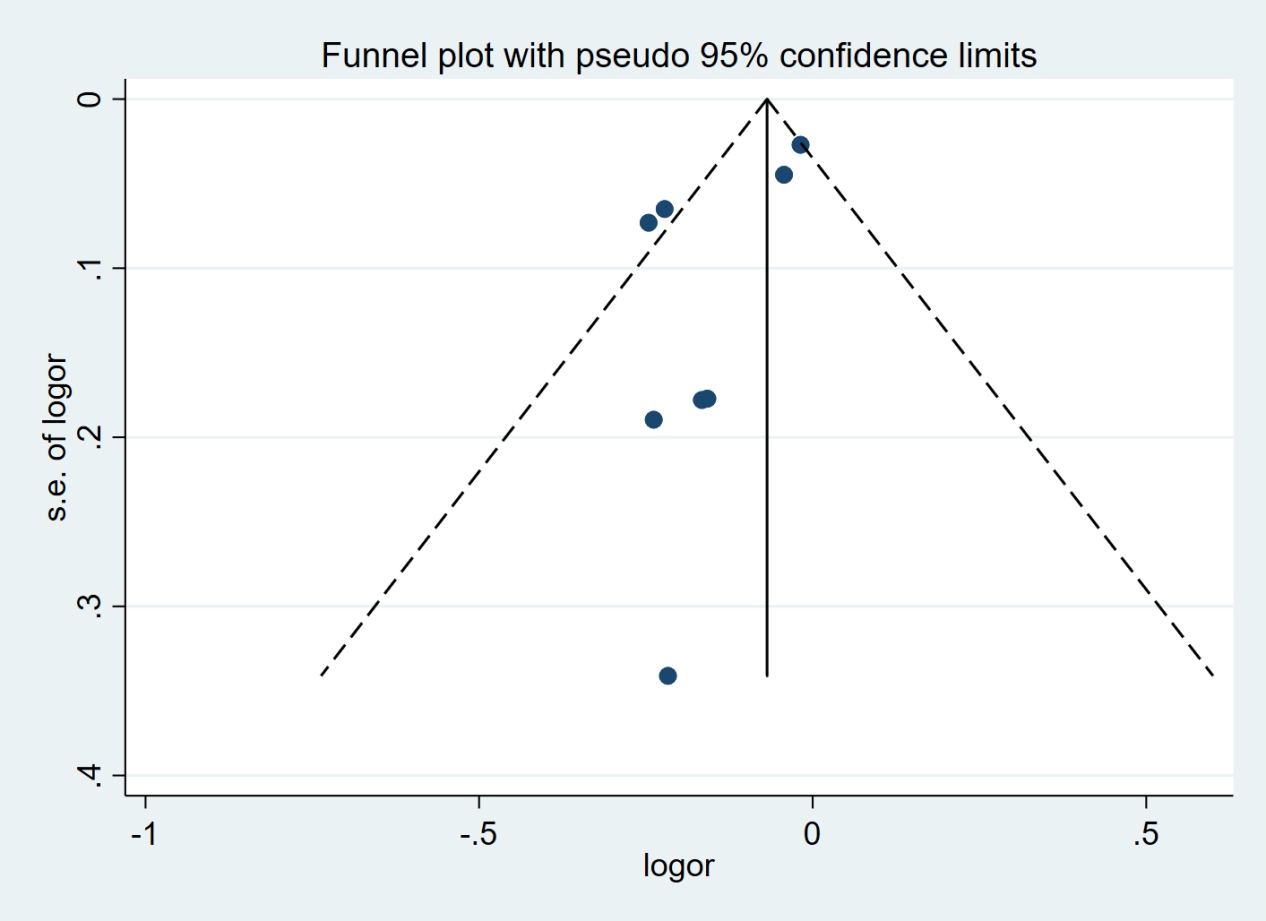

Supplement: Supplementary file 1 [file Table_1.docx]
